# Multidimensional Assessment of Laryngeal Risk and Quality-of-Life Impairment in Pediatric Hereditary Angioedema: A Case Report

**DOI:** 10.7759/cureus.106939

**Published:** 2026-04-13

**Authors:** Mokhigul Ismatova, Nodira Zakhidova, Linara Rumi, Bakhrom Mamajanov

**Affiliations:** 1 Allergy and Immunology, International Allergy Center, Tashkent, UZB; 2 Allergy, International Allergy Center, Tashkent, UZB

**Keywords:** aas, aect, ae-qol, hereditary angioedema, low-frequency phenotype

## Abstract

Hereditary angioedema (HAE) is a rare bradykinin-mediated disorder characterized by recurrent subcutaneous, abdominal, and potentially life-threatening laryngeal swelling. Disease activity is commonly monitored using the Angioedema Activity Score (AAS), although cumulative activity measures may not adequately reflect anatomical risk or imminent airway compromise. We report a pediatric patient with laryngeal HAE in whom comprehensive clinical evaluation, complement testing, and validated patient-reported outcome measures (PROMs) (AAS, Angioedema Control Test (AECT), and Angioedema Quality of Life Questionnaire (AE-QoL)) were applied. Although AAS7 and AAS28 suggested mild to low overall disease activity, the patient had recently experienced severe laryngeal edema. In contrast, a one-day AAS captured severe activity (15 points), AECT indicated markedly poor disease control (1 point), and AE-QoL demonstrated substantial quality-of-life impairment (72%), particularly within the fears/shame domain (92%). This case highlights that in high-risk HAE phenotypes, cumulative activity scores alone may underestimate true clinical risk, and that a multidimensional assessment integrating disease activity, control, anatomical localization, and quality-of-life burden is essential for accurate risk stratification and optimal management in pediatric patients.

## Introduction

Hereditary angioedema (HAE) is a rare autosomal dominant disorder most commonly caused by deficiency or dysfunction of C1 inhibitor (C1-INH), leading to uncontrolled activation of the contact system, excessive bradykinin generation, and increased vascular permeability [1,2]. The estimated prevalence is approximately 1:50,000 [1]. Clinically, HAE is characterized by recurrent, nonpruritic angioedema affecting the extremities, face, gastrointestinal tract, and upper airway; among these manifestations, laryngeal edema represents the most life-threatening complication because of the risk of asphyxiation [1].

The 2022 World Allergy Organization (WAO)/European Academy of Allergy and Clinical Immunology (EAACI) guideline underscores that the primary therapeutic objective in HAE is complete disease control and normalization of patients’ lives, including minimization of attack burden and restoration of functional capacity and psychological security [1]. For structured monitoring, validated patient-reported outcome measures (PROMs) are recommended, including the Angioedema Activity Score (AAS) to assess disease activity, the Angioedema Control Test (AECT) to evaluate disease control, and the Angioedema Quality of Life Questionnaire (AE-QoL) to measure disease-specific quality-of-life impairment [1,3-5]. However, clinically meaningful discordance may arise between cumulative “activity” scores and actual short-term risk, particularly when a single severe laryngeal attack occurs in a patient with otherwise low attack frequency. This discrepancy highlights an important gap in risk stratification and forms the clinical rationale for therapeutic decision-making and long-term prophylaxis strategies.

## Case presentation

**Figure 1:**
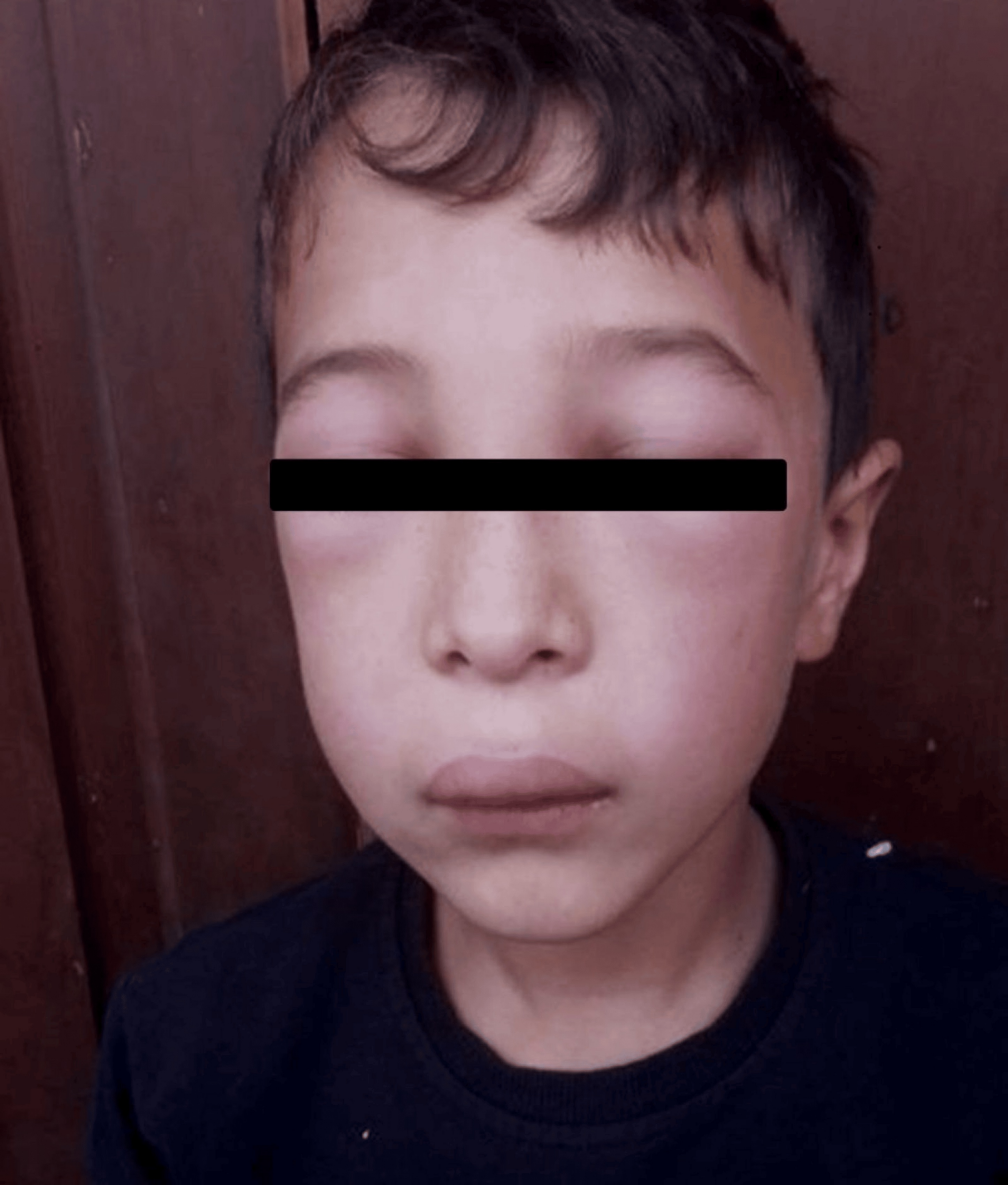
Photograph of the patient before hospitalization

**Figure 2:**
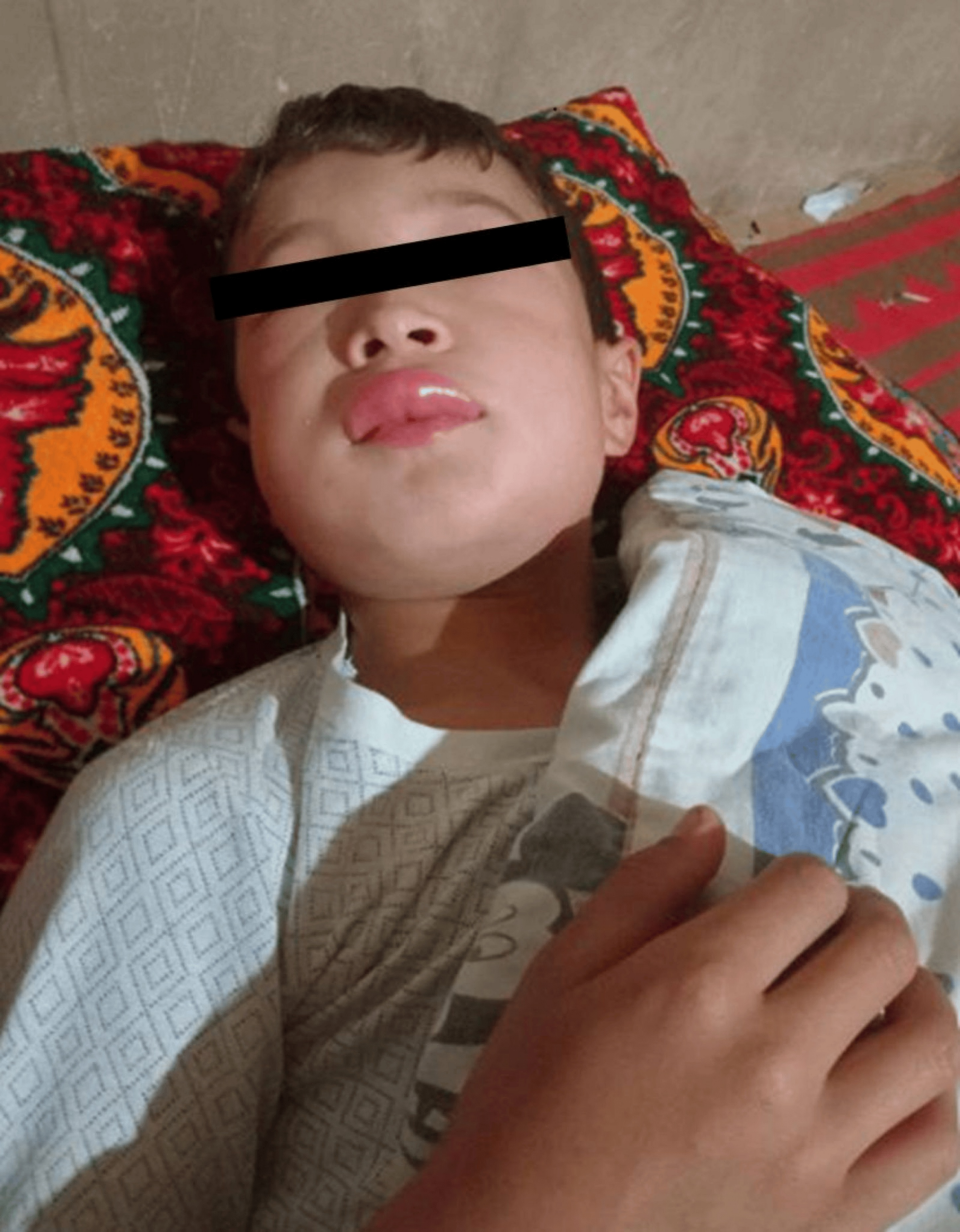
Photo of the patient before hospitalization.

An 11-year-old boy had a prior history of a low-frequency phenotype characterized by recurrent swelling of the forearms/hands, lower legs/feet, or face, as well as episodic abdominal pain with vomiting and diarrhea. Attack frequency was approximately once every 1-3 months, followed by symptom-free intervals. Several days before presentation, the patient developed facial swelling (Figure 1) and laryngeal edema (Figure 2). He was hospitalized and treated with tranexamic acid (25 mg/kg) and fresh frozen plasma, as Icatibant (a bradykinin B2 receptor antagonist) or Berinert (C1 inhibitor concentrate) was not available in the country, and clinical recovery occurred within one day (Table 1). Past surgical history included neonatal intestinal obstruction surgery (at 20 days of age), inguinal hernia repair (at 6 years), and reconstructive surgery after ulnar fracture (at 9 years). According to his anamnesis, evident swelling began at age 6 years, was migratory, nonpruritic, associated with paresthesia, and resolved spontaneously. No benefit from antihistamines or systemic corticosteroids was reported. Family history was notable for recurrent swelling episodes in the patient’s grandmother. Laboratory tests showed normal CBC and total IgE, with low C4 (0.03 g/L; reference, 0.1-0.4 g/L) on repeated testing. Unfortunately, C1-INH level/function was not measured because diagnostic testing for C1-INH level and activity was not available in the country. Based on phenotype, family history, and persistently low C4, a working diagnosis of HAE was made, consistent with guideline diagnostic principles [1,2]. Persistently low C4 in the presence of a compatible clinical phenotype strongly suggests C1-INH-related HAE; however, the absence of C1-INH antigenic and functional measurements precludes definitive subtype classification.

**Table 1:** Management and follow-up timeline. HAE: Hereditary angioedema; LTP: Long-term prophylaxis; QoL: Quality of life.

Timepoint	Clinical Event	Intervention	Outcome
Age 6	First edema episodes	None	Spontaneous resolution
Age 6-11	Recurrent attacks	Antihistamines/steroids (ineffective)	Intermittent disease
Pre-presentation	Laryngeal edema	Emergency care	High-risk event
Initial visit	Diagnosis of HAE	Tranexamic acid	Rapid improvement
Follow-up (short-term)	No attacks	Continued therapy	Stable
Future plan	Consider LTP (targeted therapy)	To be initiated	QoL improvement goal

The AAS7 (7-day cumulative score) was 15, indicating mild disease activity, while the AAS28 (28-day cumulative score) suggested minimal overall activity. However, the daily AAS reached 15 points, corresponding to severe activity on the day of the attack [3]. The AECT (4-week version) score was 1, indicating very poor disease control [4]. The AE-QoL (4-week recall) showed a total score of 72%, reflecting severe quality-of-life impairment, with a particularly pronounced impact in the fears/shame domain (92%) [5,6]. Together, these findings demonstrate a substantial perceived disease burden and poor disease control despite low cumulative activity on AAS7 and AAS28.

## Discussion

The AAS is a validated, standardized, easy-to-use questionnaire used to determine disease activity in patients with recurrent angioedema. It can be used in patients with histamine-mediated, bradykinin-mediated, and idiopathic recurrent angioedema. The AAS is designed as a diary-type tool to be used by patients for the daily documentation of their swelling episodes, including their duration, severity, and impact on daily functioning and appearance. It consists of 5 questions with 4 answer options (scored 0-3) for each item, with a minimum score of 0 and a maximum score of 15 per day. Lower and higher scores represent low and high disease activity, respectively. The AAS7 and AAS28 are the AAS measures that describe disease activity over 7 and 28 consecutive days, respectively. The AAS over 7 days (AAS7) is a validated, prospective patient-reported outcome developed to quantify short-term angioedema activity by integrating daily frequency, duration, and symptom intensity into a cumulative weekly score (0-105). The AAS28 represents the sum of the daily scores over 28 consecutive days, resulting in a possible range of 0-420 points. The reliability and construct validity of the AAS have been demonstrated in recurrent angioedema populations, and it is widely used for monitoring disease activity and treatment response [3]. In HAE, clinical danger is driven primarily by attack location rather than cumulative activity burden. Laryngeal edema, although relatively infrequent, carries an immediate risk of asphyxiation and remains a major cause of mortality in untreated patients [1,2]. Consequently, a patient may present with a low AAS7 consistent with “mild activity,” yet experience a single laryngeal episode requiring urgent on-demand therapy. This illustrates a clinically relevant activity-risk discordance, in which aggregated weekly symptom scoring underestimates life-threatening potential.

The abrupt onset and unpredictable localization of bradykinin-driven mucosal swelling are not linearly correlated with preceding inflammatory escalation or weekly symptom frequency. Because AAS7 captures symptom intensity and impact but does not weight airway involvement, progression velocity, or the need for emergency intervention, it may incompletely reflect true clinical risk. This limitation is particularly relevant in pediatric HAE, where communication barriers, caregiver burden, and heightened anxiety may amplify the clinical significance of even isolated high-risk attacks [2]. Current international HAE guidelines emphasize individualized risk assessment, including prior laryngeal involvement, when determining indications for long-term prophylaxis, rather than relying solely on attack frequency metrics [1].

This case highlights a clinically important distinction: attack activity (how often, how much swelling) can diverge from risk (probability of catastrophic outcome). Patients with airway involvement require careful reassessment of management strategy and readiness for escalation, even if attacks appear infrequent [1]. In practical terms, a low-frequency phenotype can still be a high-risk phenotype when the airway is involved.

Value of multidimensional assessment

Discordance between AAS7 and AAS28 and the short-horizon tool Daily AAS supports a multidimensional approach that integrates: activity (AAS) [3], control (AECT, including the validated cutoff of ≥10 for well-controlled disease) [4], impact/QoL (AE-QoL) [5], and clinical/anatomical risk history (laryngeal edema) [1,2]. The AE-QoL (4-week recall) captured substantial impairment of 72%, particularly in the fear/shame domain (92%), which is especially relevant in airway-risk patients, in whom fear of suffocation may persist between attacks and is not necessarily proportional to attack counts [5,6].

Therapeutic implications and modern targeted prophylaxis

While tranexamic acid may provide benefit in some settings, current international guidance prioritizes effective, targeted approaches to achieve sustained control and normalize life [1]. Modern long-term prophylaxis options demonstrate that stable control is associated with meaningful improvements in QoL and reductions in anticipatory anxiety and fear: Lanadelumab (anti-plasma kallikrein): real-world effectiveness data show strong attack suppression and improvements in disease control and QoL [7]; Berotralstat (oral kallikrein inhibitor): long-term results demonstrate sustained reductions in attacks, with improvements in AE-QoL over extended follow-up [8]; Donidalorsen (prekallikrein antisense): phase 3 data show reduced attack rates and improved patient-reported outcomes, including QoL and control, reinforcing the linkage between biochemical pathway suppression and lived benefit [9].

Taken together, these data support that in patients with prior laryngeal edema, the therapeutic target should be not only fewer attacks, but also psychological safety, elimination of fear of suffocation, and restoration of daily functioning, consistent with guideline goals [1]. Finally, we can conclude that although AAS remains an essential instrument for quantifying disease activity, activity does not equate to risk in HAE. Multidimensional evaluation incorporating attack localization, emergency treatment use, patient-reported control, and assessment of quality of life is necessary to avoid underestimation of severity and to guide optimal prophylactic management.

## Conclusions

PROMs should be integrated with anatomical risk assessment and clinical history, as “low activity” scores (AAS7/AAS28) may coexist with high airway risk and significant clinical danger in life-threatening HAE phenotypes characterized by infrequent but high-risk laryngeal attacks. Accordingly, a multidimensional approach, combining validated PROMs with anatomical risk stratification, is essential to guide therapeutic decision-making. Modern targeted therapies can achieve sustained disease control and substantially improve quality of life and psychological safety, which are critical outcomes in patients with airway-risk HAE.
